# Dietary protein intake, animal-to-plant protein ratio and risk of sarcopenia in community-dwelling adults: a 9-year follow-up study

**DOI:** 10.1017/S1368980026102973

**Published:** 2026-07-02

**Authors:** Tomoyo Komata, Keiko Kabasawa, Kaori Kitamura, Yumi Watanabe, Yumi Ito, Akemi Takahashi, Toshiko Saito, Junta Tanaka, Kei Watanabe, Ribeka Takachi, Shoichiro Tsugane, Suguru Yamamoto, Kazutoshi Nakamura

**Affiliations:** 1 Division of Preventive Medicine, Niigata University Graduate School of Medical and Dental Sciences, Niigata, Japan; 2 Department of Health Promotion Medicine, Niigata University Graduate School of Medical and Dental Sciences, Niigata, Japan; 3 Department of Rehabilitation, Niigata University of Rehabilitation, Niigata, Japan; 4 Health and Wellness Center, University of Niigata Prefecture, Niigata, Japan; 5 Department of Orthopedic Surgery, Niigata University Medical and Dental Hospital, Niigata, Japan; 6 Department of Food Science and Nutrition, Nara Women’s University Graduate School of Humanities and Sciences, Nara, Japan; 7 Department of Food Science and Nutrition, International University of Health and Welfare Graduate School of Public Health, Tokyo, Japan; 8 Division of Clinical Nephrology and Rheumatology, Kidney Research Center, Niigata University Graduate School of Medical and Dental Sciences, Niigata, Japan

**Keywords:** Animal protein intake, Animal-to-plant protein ratio, Cohort study, Plant protein intake, Sarcopenia

## Abstract

**Objective:**

This study aimed to examine the association between protein intake and risk of sarcopenia, with a specific focus on protein sources, in middle-aged and older adults.

**Design:**

This was a 9-year follow-up study. Dietary protein intake was assessed by a validated FFQ with energy adjustment using the residual method. Sarcopenia was defined by using height-adjusted appendicular lean mass and grip strength. Adjusted OR for sarcopenia were calculated by multivariable logistic regression analysis.

**Setting:**

Baseline surveys (2011–2014) for the Murakami and Uonuma cohorts assessed body size, lifestyle and medical history. Sarcopenia-related measurements were conducted in 2021–2022 in Murakami, Sekikawa, Uonuma and Minamiuonuma, Japan.

**Participants:**

6232 individuals aged 40–74 years (mean age, 62·4 years; females, 54·8 %).

**Results:**

An inverse association between plant protein intake and sarcopenia was observed in males (*P*_for trend_ = 0·018), although total and animal protein intakes were not. Animal-to-plant protein ratio was U-shaped in its association with sarcopenia; adjusted OR for the lowest and highest quintiles of the ratio compared to the middle quintile were 1·71 (95 % CI: 0·96, 3·07) and 1·97 (95 % CI: 1·10, 3·53), respectively, for males (*P*_for quadratic term_ = 0·017), and 1·93 (95 % CI: 1·06, 3·51) and 1·65 (95 % CI: 0·90, 3·05), respectively, for females (*P*_for quadratic term_ = 0·010).

**Conclusions:**

An imbalanced intake of plant and animal proteins may increase the risk of sarcopenia, highlighting the importance of balanced protein intake.

Sarcopenia is a progressive and generalised skeletal muscle disorder that involves the accelerated loss of muscle mass and function^(1)^. Sarcopenia is associated with increased adverse physical outcomes, including physical frailty^(2)^, poor prognosis in patients with chronic diseases^(3,4)^ and mortality^(5)^. The prevalence of sarcopenia is high, ranging from 10 % to 27 % using different classifications and cut-off points^(6)^. The prevalence increases as age increases, with the highest prevalence observed in individuals aged ≥ 85 years according to studies in East Asia involving older adults^(7)^. However, the presence of sex differences is unclear and may depend on the diagnostic criteria applied^(6)^. Direct and indirect medical costs associated with sarcopenia are also high. In the USA, direct medical costs of sarcopenia were estimated to be $18·5 billion in 2000^(8)^. Preventing sarcopenia not only improves individual health but also has the potential to reduce healthcare costs^(9)^.

Nutrition is a key factor in sarcopenia. Among macronutrients, protein has been extensively studied because it is a source of amino acids and an anabolic stimulus for muscle protein synthesis^(10)^. However, there is ongoing debate over whether high dietary protein intake in older adults is effective in preventing sarcopenia^(11)^. A recent systematic review reported that 18 of 26 analyses from seventeen studies (eleven cross-sectional and six follow-up studies) found a significant positive association between dietary protein intake and skeletal muscle mass in older individuals^(12)^. Among the six studies, only three employed an energy-adjusted method to assess protein intake^(12)^. Moreover, few follow-up studies have used sarcopenia as an outcome, and only one population-based follow-up study has been published^(13)^. Additional well-designed follow-up studies are needed to further elucidate the impact of habitual protein intake on sarcopenia risk.

Previous studies have reported that the intake of animal protein and plant protein, as well as their balance, influences the risk of type 2 diabetes, all-cause mortality and CVD mortality^(14,15)^. This suggests that different protein sources (animal *v*. plant protein) and their intake ratios may have distinct effects on muscle loss and the development of sarcopenia^(13,16,17)^. Differences in protein sources should also be considered when assessing protein intake in relation to sarcopenia.

The aim of this follow-up study was to examine the association between habitual protein intake and risk of sarcopenia in middle-aged and older adults, with a specific focus on animal protein intake, plant protein intake and animal-to-plant protein ratio as predictors.

## Methods

### Study setting and participants

This was a 9-year follow-up study using subsamples of two population-based cohorts, the Murakami cohort^(18)^ (*n* 14 364, 2011) and the Uonuma cohort^(19)^ (*n* 39 763, 2012) in Japan. Baseline surveys for the Murakami and Uonuma cohort studies were conducted in 2011–2013 and 2012–2014, respectively, to assess body size, lifestyle including dietary intake and medical history. Body composition measurements to assess the status of sarcopenia were conducted in 2021–2022 at local government health checkups in Sekikawa Village and Murakami City for the Murakami cohort and Uonuma City and Minamiuonuma City for the Uonuma cohort. Given the comparable design and population characteristics, we combined the two cohorts and conducted the analyses using the pooled dataset.

**Figure 1.**
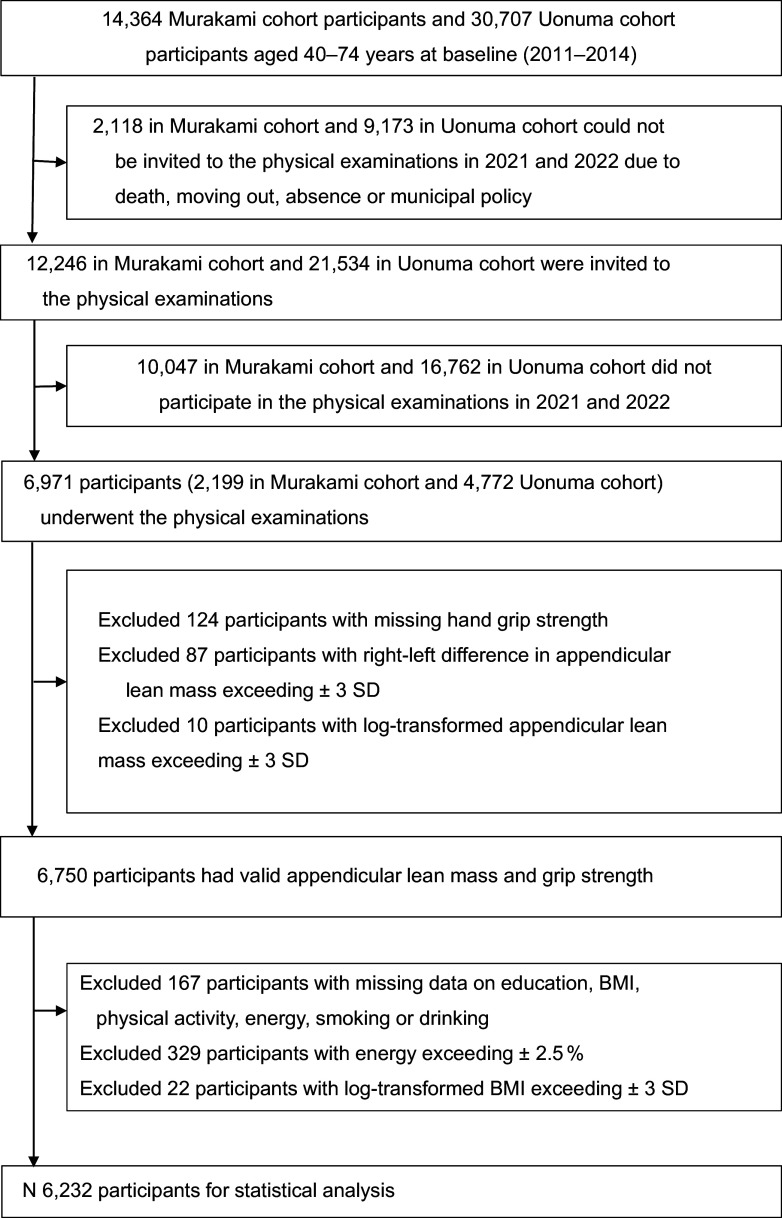
Flow chart of participant selection.

The flow of participant selection for statistical analyses is shown in Figure 1. For this study, 12 246 individuals of the Murakami cohort and 21 534 individuals of the Uonuma cohort were invited to participate in the 2021–2022 body composition examination by postal mail. A total of 6971 participants (2199 from the Murakami cohort and 4772 from the Uonuma cohort) attended. We excluded participants with a left-right difference in appendicular lean mass (ALM) exceeding ± 3 sd due to the potential presence of hemiplegia or residual metal in the body. We further excluded those with missing questionnaire data, those with mean ± 3 sd in BMI and those in the upper or lower 2·5 percentile of energy intake, leaving 6232 individuals as the analytic population.

### Dietary assessment

Dietary intake, including protein intake, was assessed using a previously validated semi-quantitative FFQ^(20)^ based on the Japanese Standard Tables of Food Composition 2010^(21)^. We assessed total, animal and plant protein intakes. Meat, processed meats, fish, eggs, milk and dairy products were classified as animal protein sources, while legumes, grains, vegetables, fruits and other food items (e.g. sweets and seasonings) were classified as plant sources^(22)^. The validity of nutrient intake assessed by the FFQ was evaluated using 12-d dietary records^(20)^. Spearman’s correlation coefficients between energy-adjusted protein intake from the FFQ and dietary records were as follows: 0·40 (males) and 0·33 (females) for total protein^(20)^; 0·34 (males) and 0·23 (females) for animal protein; 0·55 (males) and 0·47 (females) for plant protein and 0·51 (males) and 0·33 (females) for animal-to-plant protein ratio (values other than total protein are unpublished data^(20)^). Energy-adjusted total, animal and plant protein intakes were calculated by sex using the residual method^(23)^.

### Sarcopenia

For body composition measurements, ALM was estimated using multi-frequency bioelectrical impedance analysis (MF-BIA) with a standing 8-electrode MF-BIA device (MC-780A-N, TANITA). The equation used to estimate ALM in this model has been published, and a previous validation study reported a high correlation (*r* ≈ 0·92–0·93) between ALM estimated by MF-BIA and that measured by dual-energy X-ray absorptiometry^(24)^. Grip strength was measured twice for both hands using a digital hand dynamometer (Grip-D, TKK-5401; Takei Scientific Instruments Co., Ltd.). The highest value of the two measurements for each hand was used for statistical analysis. Sarcopenia was defined based on adjusted ALM (ALM (kg)/height^2^ (m^2^)) and grip strength following the Asian Working Group for Sarcopenia (AWGS) 2019^(25)^. However, using these criteria, the prevalence of sarcopenia was particularly low, especially in females^(26)^. To address this issue, Yamada *et al.* ^(26)^ proposed and validated an alternative cut-off value for sarcopenia in older adults, defined as the 20th percentile of the adjusted ALM. Following this approach, we determined cut-off values of the adjusted ALM of < 7·0 kg/m^2^ for males and < 6·1 kg/m^2^ for females. The cut-off values of grip strength for defining sarcopenia were set at 28 kg for males and 18 kg for females^(25)^. Participants were classified as having sarcopenia if they met both criteria of low adjusted ALM and low grip strength based on these cut-off values.

### Covariates

Murakami and Uonuma cohort studies used the same self-reported questionnaire which collected information on demographics, body size^(27)^, smoking^(28)^, alcohol consumption^(29)^, physical activity (PA)^(30)^ and medical history. Codes for demographic variables, smoking and alcohol consumption are shown in online supplementary material, Supplemental Table 1. PA levels were assessed using metabolic equivalent (MET) hours per day (MET score) based on the Japanese Public Health Center-based Prospective Study Physical Activity Questionnaire^(31)^. Height and weight were self-reported, and BMI was calculated by dividing weight (kg) by height squared (m^2^). We verified self-reported height and body weight (BW) against precise anthropometric measurements, yielding intra-class correlation coefficients of 0·985 for height and 0·983 for weight^(32)^. Regarding medical history, stroke^(33)^, diabetes^(34)^ and heart disease (myocardial infarction and heart failure)^(35)^ have been reported in previous studies as independent risk factors for sarcopenia. Therefore, we considered these conditions potential confounders and included them as covariates in this study.

### Statistical analysis

Continuous variables for participant characteristics are presented as median and interquartile range by quartiles of protein intake. Animal-to-plant protein ratio was classified into quintiles given the possibility that the association between the ratio and health outcomes follows a U-shaped pattern^(15)^. *P*_for trend_ values for participant characteristics were calculated using ordinal logistic regression analysis. The outcome was sarcopenia, and predictors were total protein intake, animal protein intake, plant protein intake and animal-to-plant protein ratio. Logistic regression analysis was used to calculate OR and 95 % CI for sarcopenia. We ran three models: unadjusted, age-adjusted and multivariable-adjusted models. The multivariable logistic regression model included age, marital status (dummy variable), education, occupation (dummy variable), log-transformed BMI, total PA, smoking, alcohol consumption and history of stroke, diabetes and heart disease as covariates. It is debatable, however, whether BMI serves as an independent risk factor, given that BMI is likely a mediator in the association between energy intake and sarcopenia and a potential mediator in the association between protein intake and sarcopenia. To address this issue, we presented two multivariable-adjusted models (model 1 excluding BMI and model 2 including BMI). *P*_for trend_ values were calculated using logistic regression analysis. *P*-values for quadratic terms were calculated to assess the U-shaped association between quintiles of animal-to-plant protein ratio and odds of sarcopenia. SAS statistical software (Release 9.4, SAS Institute Inc.) was used for statistical analysis.

## Results

We first compared the basic characteristics of the present subsample with those of the original total sample of the Murakami/Uonuma cohorts (see online supplementary material, Supplemental Table 2). Older individuals, females, those with a junior high school education and those without an occupation were more likely to participate in the 2021–2022 examinations.

The mean age of participants of the present study at baseline was 62·4 years, and the mean follow-up period was 9·1 years (sd, 1·6). Participant characteristics at baseline according to quartiles of total, animal and plant protein intakes are shown in Table 1 for males and Table 2 for females. Males with higher total protein intake tended to be older, have lower PA levels, have higher animal and plant protein intakes, be more likely to have a university degree and be less likely to be manual workers, smokers or drinkers. Females with higher total protein intake tended to be more likely to have a history of diabetes, have lower PA levels, have higher plant protein intake and be more likely to have a university degree; other characteristics were similar to those of males. Males with higher animal protein intake tended to be older, have lower PA levels, have higher total protein but lower plant protein and energy intakes, be more likely to have a university degree, be more likely to have a history of diabetes and be less likely to be manual workers, smokers or drinkers. Females with higher animal protein intake tended to have lower PA levels, have lower energy intake, be more likely to have a university degree and be more likely to have a history of diabetes; other characteristics were similar to those of males. Males with higher plant protein intake tended to be older, have higher total protein intake and lower animal protein intake and be less likely to be smokers or drinkers. Females with higher plant protein intake tended to have higher PA levels, have higher energy and total protein intakes, be more likely to be married and be less likely to be smokers; other characteristics were similar to those of males. Participant characteristics at baseline according to quartiles of total, animal and plant protein intakes for males and females combined are shown in online supplementary material, Supplemental Table 3. Participant characteristics at baseline according to quintiles of animal-to-plant protein ratio are shown in online supplementary material, Supplemental Table 4. At the 9-year follow-up, sarcopenia was observed in 4·8 % and 3·6 % of males and females, respectively (see online supplementary material, Supplemental Table 5).

**Table 1.** Participant characteristics at the 2011–2014 baseline survey according to quartiles of total, animal and plant protein intakes in males Table 1 long description.

	Quartiles of energy-adjusted intake								*P*_for trend_
	Q1		Q2		Q3		Q4		
**Total protein**									
*n*	703		705		704		703		
	Median	IQR	Median	IQR	Median	IQR	Median	IQR	
Age (years)	63·0	58·0–67·0	64·0	60·0–68·0	65·0	60·0–69·0	65·0	60·0–69·0	< 0·001
BMI (kg/m^2^)	22·9	21·1–24·4	22·9	21·4–24·6	22·9	21·2–24·3	23·1	21·2–24·8	0·24
Total PA (MET-h/d)	43·6	35·8–54·0	42·2	35·5–51·9	41·7	35·1–52·8	40·1	34·9–50·7	0·002
Energy (kcal/d)	2136	1734–2547	2230	1832–2646	2132	1772–2624	2089	1703–2546	0·13
Total protein (g/d)	51·3	39·3–63·5	66·7	52·2–82·3	69·7	56·3–90·4	79·3	63·7–104·8	< 0·001
Animal protein (g/d)	17·4	11·0–25·1	27·2	19·1–38·3	32·3	23·1–46·8	42·9	30·1–62·3	< 0·001
Plant protein (g/d)	32·4	25·6–40·7	36·7	30·1–45·7	37·2	29·8–44·5	33·9	28·4–43·5	< 0·001
	*n*	%	*n*	%	*n*	%	*n*	%	
Married (%)	569	80·9 %	620	87·9 %	603	85·7 %	569	80·9 %	0·77
University graduates (%)	38	5·4 %	58	8·2 %	80	11·4 %	86	12·2 %	< 0·001
Manual job (%)	269	38·3 %	242	34·3 %	247	35·1 %	185	26·3 %	< 0·001
Current smoker (%)	225	32·0 %	175	24·8 %	151	21·5 %	150	21·3 %	< 0·001
Current drinker (%)	657	93·5 %	607	86·1 %	566	80·4 %	495	70·4 %	< 0·001
History of stroke (%)	13	1·9 %	19	2·7 %	24	3·4 %	20	2·8 %	0·2
History of diabetes (%)	51	7·3 %	62	8·8 %	54	7·7 %	74	10·5 %	0·06
History of heart disease (%)	6	0·9 %	10	1·4 %	12	1·7 %	7	1·0 %	0·73
**Animal protein**									
*n*	703		705		704		703		
Age (years)	63·0	59·0–68·0	64·0	60·0–68·0	64·0	60·0–68·0	64·0	59·0–69·0	< 0·001
BMI (kg/m^2^)	22·9	21·3–24·8	22·9	21·5–24·4	22·9	21·1–24·5	23·0	21·0–24·7	0·7
Total PA (MET-h/d)	43·2	35·7–53·2	43·0	36·0–53·8	40·9	35·1–51·2	40·3	34·7–51·4	0·005
Energy (kcal/d)	2183	1743–2559	2236	1842–2675	2164	1813–2613	2010	1657–2494	< 0·001
Total protein (g/d)	54·8	41·2–68·9	66·7	52·0–84·2	69·7	56·3–88·4	74·2	57·6–99·8	< 0·001
Animal protein (g/d)	15·4	9·2–22·3	27·2	19·7–37·3	34·3	25·2–47·9	43·6	31·1–63·3	< 0·001
Plant protein (g/d)	38·8	30·1–48·4	38·6	31·1–46·5	35·7	29·2–41·7	30·2	24·7–35·8	< 0·001
	*n*	%	*n*	%	*n*	%	*n*	%	
Married (%)	577	82·1 %	608	86·2 %	600	85·2 %	576	81·9 %	0·82
University graduates (%)	43	6·1 %	54	7·7 %	85	12·1 %	80	11·4 %	< 0·001
Manual job (%)	242	34·4 %	275	39·0 %	233	33·1 %	193	27·5 %	< 0·001
Current smoker (%)	207	29·5 %	183	26·0 %	147	20·9 %	164	23·3 %	0·001
Current drinker (%)	616	87·6 %	589	83·6 %	589	83·7 %	531	75·5 %	< 0·001
History of stroke (%)	21	3·0 %	19	2·7 %	15	2·1 %	21	3·0 %	0·83
History of diabetes (%)	52	7·4 %	56	7·9 %	53	7·5 %	80	11·4 %	0·012
History of heart disease (%)	8	1·1 %	7	1·0 %	10	1·4 %	10	1·4 %	0·49
**Plant protein**									
*n*	703		705		703		704		
Age (years)	63·0	58·0–67·0	64·0	59·0–68·0	64·0	60·0–69·0	64·0	60·0–69·0	< 0·001
BMI (kg/m^2^)	22·8	21·1–24·4	23·1	21·3–24·7	22·9	21·3–24·6	22·9	21·2–24·7	0·46
Total PA (MET-h/d)	41·9	34·9–53·4	41·5	34·9–52·4	42·5	35·7–52·2	41·9	35·5–51·4	0·52
Energy (kcal/d)	2186	1750–2682	2081	1740–2497	2160	1784–2582	2181	1790–2627	0·48
Total protein (g/d)	63·4	46·1–89·3	63·1	48·9–78·9	66·2	51·6–85·1	70·5	56·4–89·1	< 0·001
Animal protein (g/d)	35·3	21·5–58·1	30·2	20·3–43·9	28·3	17·9–40·7	24·2	16·4–34·3	< 0·001
Plant protein (g/d)	27·3	22·1–32·9	32·3	28·0–38·2	38·3	32·5–44·7	45·8	38·4–55·1	< 0·001
	*n*	%	*n*	%	*n*	%	*n*	%	
Married (%)	576	81·9 %	601	85·2 %	596	84·8 %	588	83·5 %	0·46
University graduates (%)	63	9·0 %	59	8·4 %	74	10·5 %	66	9·4 %	0·49
Manual job (%)	225	32·0 %	235	33·3 %	257	36·6 %	226	32·1 %	0·72
Current smoker (%)	214	30·4 %	184	26·1 %	152	21·6 %	151	21·5 %	< 0·001
Current drinker (%)	649	92·3 %	591	83·8 %	570	81·1 %	515	73·2 %	< 0·001
History of stroke (%)	14	2·0 %	19	2·7 %	18	2·6 %	25	3·6 %	0·09
History of diabetes (%)	63	9·0 %	62	8·8 %	56	8·0 %	60	8·5 %	0·65
History of heart disease (%)	10	1·4 %	7	1·0 %	10	1·4 %	8	1·1 %	0·82

PA, physical activity; MET, metabolic equivalent.

Data are presented as the median and interquartile range or number. BMI, energy intake, total protein intake, animal protein intake and plant protein intake were log-transformed when calculating *P*_for trend_.

**Table 2.** Participant characteristics at the 2011–2014 baseline survey according to quartiles of total, animal and plant protein intakes in females Table 2 long description.

	Quartiles of energy-adjusted intake								*P*_for trend_
	Q1		Q2		Q3		Q4		
**Total protein**									
*n*	855		854		854		854		
	Median	IQR	Median	IQR	Median	IQR	Median	IQR	
Age (years)	61·0	56·0–66·0	62·0	58·0–67·0	63·0	58·0–67·0	64·0	60·0–68·0	< 0·001
BMI (kg/m^2^)	21·8	20·0–23·9	22·0	20·3–23·8	22·1	20·4–23·9	22·1	20·3–24·1	0·27
Total PA (MET-h/d)	39·0	35·0–47·7	39·0	34·9–45·9	39·4	35·2–46·4	39·0	35·1–45·6	0·37
Energy (kcal/d)	1856	1495–2306	1863	1532–2312	1854	1543–2232	1860	1486–2333	0·97
Total protein (g/d)	55·0	43·0–71·8	63·7	50·8–82·5	69·2	55·8–86·9	79·8	61·5–106·4	< 0·001
Animal protein (g/d)	20·8	14·2–29·4	28·5	20·4–39·3	34·3	25·2–46·1	43·9	31·0–65·1	< 0·001
Plant protein (g/d)	33·5	26·8–42·8	35·3	28·6–43·5	34·1	28·3–41·9	32·9	26·8–40·9	0·12
	*n*	%	*n*	%	*n*	%	*n*	%	
Married (%)	704	82·3 %	692	81·0 %	715	83·7 %	709	83·0 %	0·43
University graduates (%)	20	2·3 %	23	2·7 %	25	2·9 %	23	2·7 %	0·61
Manual job (%)	129	15·1 %	108	12·6 %	114	13·3 %	89	10·4 %	0·009
Current smoker (%)	54	6·3 %	31	3·6 %	33	3·9 %	32	3·8 %	0·012
Current drinker (%)	405	47·4 %	348	40·8 %	335	39·2 %	295	34·5 %	< 0·001
History of stroke (%)	7	0·8 %	10	1·2 %	11	1·3 %	15	1·8 %	0·09
History of diabetes (%)	25	2·9 %	30	3·5 %	29	3·4 %	45	5·3 %	0·015
History of heart disease (%)	0	0·0 %	1	0·1 %	1	0·1 %	0	0·0 %	1
**Animal protein**									
*n*	854		855		854		854		
Age (years)	62·0	57·0–66·0	62·0	57·0–66·0	63·0	58·0–67·0	64·0	59·0–67·0	< 0·001
BMI (kg/m^2^)	21·9	20·1–24·0	22·0	20·2–23·8	21·9	20·3–23·8	22·2	20·3–24·1	0·14
Total PA (MET-h/d)	39·4	34·9–47·3	39·1	35·2–46·3	39·3	35·3–46·2	38·9	34·8–45·7	0·09
Energy (kcal/d)	1824	1489–2312	1864	1556–2299	1873	1544–2272	1826	1480–2327	0·99
Total protein (g/d)	56·8	43·7–74·7	63·9	51·2–81·7	68·7	55·8–87·3	77·0	59·4–104·2	< 0·001
Animal protein (g/d)	18·1	12·7–25·6	28·2	21·4–37·6	35·4	26·4–47·1	46·8	33·4–67·3	< 0·001
Plant protein (g/d)	38·1	30·4–48·5	35·9	29·6–44·0	33·5	28·3–40·0	29·4	24·0–35·4	< 0·001
	*n*	%	*n*	%	*n*	%	*n*	%	
Married (%)	703	82·3 %	714	83·5 %	692	81·0 %	711	83·3 %	0·88
University graduates (%)	13	1·5 %	23	2·7 %	34	4·0 %	21	2·5 %	0·13
Manual job (%)	127	14·9 %	108	12·6 %	118	13·8 %	87	10·2 %	0·013
Current smoker (%)	53	6·2 %	32	3·7 %	31	3·6 %	34	4·0 %	0·022
Current drinker (%)	358	41·9 %	364	42·6 %	337	39·5 %	324	37·9 %	0·045
History of stroke (%)	10	1·2 %	7	0·8 %	11	1·3 %	15	1·8 %	0·17
History of diabetes (%)	33	3·9 %	24	2·8 %	30	3·5 %	42	4·9 %	0·16
History of heart disease (%)	0	0·0 %	0	0·0 %	1	0·1 %	1	0·1 %	0·29
**Plant protein**									
*n*	854		855		854		854		
Age (years)	62·0	57·0–66·0	62·0	58·0–66·0	63·0	59·0–67·0	63·0	59·0–67·0	< 0·001
BMI (kg/m^2^)	22·1	20·3–24·0	22·0	20·3–24·0	21·9	20·1–23·9	21·9	20·3–23·9	0·09
Total PA (MET-h/d)	39·0	34·9–46·4	38·4	34·8–45·5	39·3	35·2–45·9	40·1	35·6–47·6	0·042
Energy (kcal/d)	1841	1489–2370	1810	1467–2198	1825	1511–2257	1980	1584–2372	0·004
Total protein (g/d)	67·6	50·2–95·0	64·3	49·8–81·3	64·1	51·5–83·5	71·3	55·9–89·1	0·07
Animal protein (g/d)	40·1	26·2–62·5	32·3	22·1–44·3	28·9	19·9–41·3	25·6	17·3–35·9	< 0·001
Plant protein (g/d)	26·8	21·8–32·4	31·5	26·8–37·6	35·8	30·4–42·9	44·2	36·6 52·9	< 0·001
	*n*	%	*n*	%	*n*	%	*n*	%	
Married (%)	685	80·2 %	718	84·0 %	700	82·0 %	717	84·0 %	0·045
University graduates (%)	30	3·5 %	22	2·6 %	21	2·5 %	18	2·1 %	0·07
Manual job (%)	112	13·1 %	119	13·9 %	97	11·4 %	112	13·1 %	0·6
Current smoker (%)	49	5·7 %	30	3·5 %	34	4·0 %	37	4·3 %	0·21
Current drinker (%)	385	45·1 %	372	43·5 %	342	40·1 %	284	33·3 %	< 0·001
History of stroke (%)	9	1·1 %	6	0·7 %	14	1·6 %	14	1·6 %	0·11
History of diabetes (%)	35	4·1 %	23	2·7 %	30	3·5 %	41	4·8 %	0·28
History of heart disease (%)	1	0·1 %	0	0·0 %	1	0·1 %	0	0·0 %	0·52

PA, physical activity; MET, metabolic equivalent.

Data are presented as the median and interquartile range or number. BMI, energy intake, total protein intake, animal protein intake and plant protein intake were log-transformed when calculating *P*_for trend_.

OR for sarcopenia according to quartiles of total protein intake are shown in Table 3. Total protein intake was not significantly associated with the multivariable-adjusted odds of sarcopenia in either sex or in the overall population. OR for sarcopenia according to quartiles of animal and plant protein intakes are shown in Table 4. Higher plant protein intake was associated with lower odds of sarcopenia (multivariable-adjusted model 2 *P*_for trend_ = 0·018), with the 3rd and 4th quartiles showing lower multivariable-adjusted model 2 OR (0·47, 95 % CI: 0·26, 0·83 and 0·53, 95 % CI: 0·29, 0·96, respectively) than the 1st quartile (reference) in males. The significant association between plant protein intake and sarcopenia was absent in the unadjusted model but emerged in the age-adjusted models, indicating confounding by age. This association was not observed in females. In the overall population, higher plant protein intake was marginally associated with lower odds of sarcopenia (multivariable-adjusted model 2 *P*_for trend_ = 0·07), with the 3rd quartile showing lower multivariable-adjusted OR (0·55, 95 % CI: 0·36, 0·83) than the reference. There was a marginally significant interaction between plant protein intake and sex on sarcopenia (multivariable-adjusted model 2 *P*_for interaction_ = 0·05). OR for sarcopenia according to quartiles of plant protein intake in participants with no disease history are shown in online supplementary material, Supplemental Table 6 (sensitivity analysis). The pattern of association was similar to that in Table 4, except the marginally significant value of *P*_for trend_ in the male multivariable-adjusted model 2 (0·08). Animal protein intake was not significantly associated with the multivariable-adjusted odds of sarcopenia in either sex or in the overall population.

**Table 3.** OR for sarcopenia according to quartiles of total protein intake Table 3 long description.

	Quartiles of energy-adjusted total protein intake								
			Q2		Q3		Q4		
	Q1		OR	95 % CI	OR	95 % CI	OR	95 % CI	*P*_for trend_
Males									
Occurrence of sarcopenia	27/703	3·8 %	37/705	5·2 %	27/704	3·8 %	44/703	6·3 %	
Unadjusted	1 (ref)		1·39	0·84, 2·30	0·999	0·58, 1·72	1·67	1·02, 2·73	0·11
Age-adjusted	1 (ref)		1·17	0·69, 1·96	0·72	0·41, 1·25	1·17	0·71, 1·95	0·9
Multivariable-adjusted model 1^*^	1 (ref)		1·20	0·69, 2·06	0·77	0·43, 1·40	1·23	0·69, 2·17	0·78
Multivariable-adjusted model 2^†^	1 (ref)		1·20	0·69, 2·09	0·72	0·39, 1·32	1·16	0·65, 2·08	0·98
Females									
Occurrence of sarcopenia	24/855	2·8 %	27/854	3·2 %	34/854	4·0 %	37/854	4·3 %	
Unadjusted	1 (ref)		1·13	0·65, 1·98	1·44	0·84, 2·44	1·57	0·93, 2·64	0·06
Age-adjusted	1 (ref)		0·96	0·55, 1·69	1·18	0·69, 2·02	1·15	0·68, 1·95	0·46
Multivariable-adjusted model 1^*^	1 (ref)		0·89	0·50, 1·57	1·10	0·64, 1·89	1·03	0·60, 1·76	0·71
Multivariable-adjusted model 2^†^	1 (ref)		0·93	0·52, 1·65	1·16	0·67, 2·02	1·08	0·62, 1·85	0·61
Males and females combined									
Occurrence of sarcopenia	51/1558	3·3 %	64/1559	4·1 %	61/1558	3·9 %	81/1557	5·2 %	
Unadjusted	1 (ref)		1·27	0·87, 1·84	1·20	0·82, 1·76	1·62	1·13, 2·32	0·013
Age-adjusted	1 (ref)		1·06	0·73, 1·56	0·93	0·63, 1·37	1·16	0·81, 1·68	0·53
Multivariable-adjusted model 1^‡^	1 (ref)		1·03	0·69, 1·52	0·91	0·61, 1·35	1·10	0·74, 1·62	0·74
Multivariable-adjusted model 2^†^	1 (ref)		1·05	0·71, 1·56	0·91	0·61, 1·36	1·11	0·75, 1·64	0·75

* Adjusted for age, marital status, education, occupation, total physical activity (PA), smoking habit, alcohol consumption and history of stroke, diabetes and heart disease.

† Further adjusted for log-transformed BMI in addition to the variables included in multivariable-adjusted model 1.

‡ Adjusted for sex, age, marital status, education, occupation, total PA, smoking habit, alcohol consumption and history of stroke, diabetes and heart disease.

**Table 4.** OR for sarcopenia according to quartiles of animal and plant protein intakes Table 4 long description.

	Quartiles of energy-adjusted protein intake								
			Q2		Q3		Q4		
	Q1		OR	95 % CI	OR	95 % CI	OR	95 % CI	*P*_for trend_
Animal protein males									
Occurrence of sarcopenia	29/703	4·1 %	33/705	4·7 %	25/704	3·6 %	48/703	6·8 %	
Unadjusted	1 (ref)		1·14	0·69, 1·90	0·86	0·50, 1·48	1·70	1·06, 2·73	0·05
Age-adjusted	1 (ref)		1·00	0·59, 1·69	0·70	0·40, 1·22	1·43	0·88, 2·33	0·23
Multivariable-adjusted model 1^*^	1 (ref)		1·00	0·59, 1·71	0·72	0·40, 1·29	1·30	0·73, 2·29	0·55
Multivariable-adjusted model 2^†^	1 (ref)		1·03	0·60, 1·76	0·67	0·37, 1·21	1·24	0·69, 2·22	0·72
Females									
Occurrence of sarcopenia	30/854	3·5 %	28/855	3·3 %	27/854	3·2 %	37/854	4·3 %	
Unadjusted	1 (ref)		0·93	0·55, 1·57	0·90	0·53, 1·52	1·24	0·76, 2·03	0·41
Age-adjusted	1 (ref)		0·94	0·55, 1·59	0·79	0·46, 1·36	1·07	0·65, 1·75	0·92
Multivariable-adjusted model 1^*^	1 (ref)		1·02	0·59, 1·76	0·89	0·50, 1·58	1·26	0·69, 2·28	0·56
Multivariable-adjusted model 2^†^	1 (ref)		0·98	0·57, 1·71	0·86	0·48, 1·54	1·26	0·69, 2·30	0·55
Males and females combined									
Occurrence of sarcopenia	59/1557	3·8 %	61/1560	3·9 %	52/1558	3·3 %	85/1557	5·5 %	
Unadjusted	1 (ref)		1·03	0·72, 1·49	0·88	0·60, 1·28	1·47	1·04, 2·06	0·049
Age-adjusted	1 (ref)		0·98	0·68, 1·42	0·75	0·51, 1·10	1·24	0·88, 1·76	0·36
Multivariable-adjusted model 1^‡^	1 (ref)		1·00	0·69, 1·47	0·78	0·52, 1·17	1·22	0·81, 1·83	0·55
Multivariable-adjusted model 2^†^	1 (ref)		0·99	0·68, 1·46	0·74	0·49, 1·12	1·20	0·79, 1·81	0·64
Plant protein males									
Occurrence of sarcopenia	39/703	5·5 %	34/705	4·8 %	27/703	3·8 %	35/704	5·0 %	
Unadjusted	1 (ref)		0·86	0·54, 1·38	0·68	0·41, 1·12	0·89	0·56, 1·42	0·45
Age-adjusted	1 (ref)		0·74	0·45, 1·21	0·48	0·29, 0·81	0·63	0·39, 1·03	0·029
Multivariable-adjusted model 1^§^	1 (ref)		0·69	0·41, 1·15	0·45	0·25, 0·79	0·54	0·30, 0·97	0·021
Multivariable-adjusted model 2^†^	1 (ref)		0·74	0·44, 1·26	0·47	0·26, 0·83	0·53	0·29, 0·96	0·018
Females									
Occurrence of sarcopenia	27/854	3·2 %	31/855	3·6 %	22/854	2·6 %	42/854	4·9 %	
Unadjusted	1 (ref)		1·15	0·68, 1·95	0·81	0·46, 1·43	1·58	0·97, 2·59	0·14
Age-adjusted	1 (ref)		1·10	0·65, 1·87	0·71	0·40, 1·27	1·36	0·83, 2·25	0·4
Multivariable-adjusted model 1^§^	1 (ref)		1·05	0·61, 1·82	0·63	0·34, 1·17	1·16	0·64, 2·09	0·95
Multivariable-adjusted model 2^†^	1 (ref)		1·01	0·58, 1·76	0·63	0·34, 1·16	1·11	0·61, 2·01	0·97
Males and females combined									
Occurrence of sarcopenia	66/1557	4·2 %	65/1560	4·2 %	49/1557	3·1 %	77/1558	4·9 %	
Unadjusted	1 (ref)		0·98	0·69, 1·39	0·73	0·50, 1·07	1·18	0·84, 1·64	0·63
Age-adjusted	1 (ref)		0·90	0·63, 1·29	0·59	0·40, 0·87	0·94	0·66, 1·32	0·37
Multivariable-adjusted model 1^‖^	1 (ref)		0·86	0·59, 1·24	0·55	0·36, 0·82	0·79	0·53, 1·18	0·1
Multivariable-adjusted model 2^†^	1 (ref)		0·87	0·59, 1·26	0·55	0·36, 0·83	0·76	0·50, 1·14	0·07

* Adjusted for age, marital status, education, occupation, energy-adjusted plant protein intake, total physical activity (PA), smoking habit, alcohol consumption and history of stroke, diabetes and heart disease.

† Further adjusted for log-transformed BMI in addition to the variables included in multivariable-adjusted model 1.

‡ Adjusted for sex, age, marital status, education, occupation, energy-adjusted plant protein intake, total PA, smoking habit, alcohol consumption and history of stroke, diabetes and heart disease.

§ Adjusted for age, marital status, education, occupation, energy-adjusted animal protein intake, total PA, smoking habit, alcohol consumption, and history of stroke, diabetes and heart disease.

‖ Adjusted for sex, age, marital status, education, occupation, energy-adjusted animal protein intake, total PA, smoking habit, alcohol consumption and history of stroke, diabetes and heart disease.

OR for sarcopenia according to quintiles of animal-to-plant protein ratio are shown in Table 5. The odds of sarcopenia were significantly associated with animal-to-plant protein ratio in a U-shaped manner in males and females (multivariable-adjusted model 2 *P*-values for quadratic term = 0·017 and 0·010, respectively), with the 5th quintile in males and the 1st quintile in females showing higher multivariable-adjusted model 2 OR (1·97, 95 % CI: 1·10, 3·53 and 1·93, 95 % CI: 1·06, 3·51, respectively) than the middle quintile (reference). There was no sex difference in the U-shaped association (multivariable-adjusted model 2 *P*_for interaction_ for the quadratic term = 0·92). Associations for the overall population were similar, with the 1st and 5th quintiles showing higher multivariable-adjusted model 2 OR (1·77, 95 % CI: 1·17, 2·68 and 1·78, 95 % CI: 1·17, 2·71, respectively) than the reference. OR for sarcopenia according to quartiles of animal-to-plant protein ratio in participants with no disease history are shown in online supplementary material, Supplemental Table 7 (sensitivity analysis). The pattern of association was similar to that in Table 5.

**Table 5.** OR for sarcopenia according to quintiles of animal-to-plant protein ratio Table 5 long description.

	Quintiles of animal-to-plant protein ratio										
	Q1		Q2				Q4		Q5		
	OR	95 % CI	OR	95 % CI	Q3		OR	95 % CI	OR	95 % CI	*P*_for quadratic term_
Males											
Occurrence of sarcopenia	32/563	5·7 %	20/563	3·6 %	23/563	4·1 %	25/563	4·4 %	35/563	6·2 %	
Unadjusted	1·42	0·82, 2·45	0·87	0·47, 1·59	1 (ref)		1·09	0·61, 1·95	1·56	0·91, 2·67	0·025
Age-adjusted	1·66	0·94, 2·91	1·08	0·58, 2·02	1 (ref)		1·28	0·71, 2·32	1·77	1·02, 3·09	0·015
Multivariable-adjusted model 1^*^	1·60	0·90, 2·83	1·14	0·60, 2·14	1 (ref)		1·34	0·74, 2·46	1·80	1·02, 3·18	0·026
Multivariable-adjusted model 2^†^	1·71	0·96, 3·07	1·28	0·67, 2·44	1 (ref)		1·42	0·77, 2·63	1·97	1·10, 3·53	0·017
Females											
Occurrence of sarcopenia	33/684	4·8 %	20/683	2·9 %	19/683	2·8 %	22/683	3·2 %	28/684	4·1 %	
Unadjusted	1·77	0·997, 3·15	1·05	0·56, 1·99	1 (ref)		1·16	0·62, 2·17	1·49	0·83, 2·70	0·022
Age-adjusted	2·00	1·11, 3·58	1·10	0·58, 2·09	1 (ref)		1·18	0·63, 2·21	1·46	0·80, 2·66	0·015
Multivariable-adjusted model 1^*^	1·95	1·08, 3·51	1·11	0·58, 2·12	1 (ref)		1·17	0·62, 2·21	1·53	0·84, 2·80	0·014
Multivariable-adjusted model 2^†^	1·93	1·06, 3·51	1·08	0·56, 2·08	1 (ref)		1·22	0·64, 2·32	1·65	0·90, 3·05	0·01
Males and females combined											
Occurrence of sarcopenia	65/1247	5·2 %	40/1246	3·2 %	42/1246	3·4 %	47/1246	3·8 %	63/1247	5·1 %	
Unadjusted	1·58	1·06, 2·34	0·95	0·61, 1·48	1 (ref)		1·12	0·74, 1·72	1·53	1·02, 2·27	0·001
Age-adjusted	1·82	1·21, 2·72	1·07	0·68, 1·68	1 (ref)		1·22	0·79, 1·87	1·60	1·06, 2·40	< 0·001
Multivariable-adjusted model 1^‡^	1·73	1·15, 2·59	1·10	0·70, 1·72	1 (ref)		1·26	0·81, 1·94	1·63	1·08, 2·46	0·001
Multivariable-adjusted model 2^†^	1·77	1·17, 2·68	1·14	0·72, 1·80	1 (ref)		1·32	0·85, 2·05	1·78	1·17, 2·71	< 0·001

Cut-off values for quintiles are 0·49, 070, 0·94 and 1·30 for males and 0·58, 0·80, 1·02 and 1·38 for females.

Median values for quintiles are 0·34, 0·60, 0·82, 1·09 and 1·67 for males and 0·44, 0·70, 0·90, 1·16 and 1·77 for females.

* Adjusted for age, marital status, education, occupation, total physical activity (PA), smoking habit, alcohol consumption and history of stroke, diabetes and heart disease.

† Further adjusted for log-transformed BMI in addition to the variables included in multivariable-adjusted model 1.

‡ Adjusted for sex, age, marital status, education, occupation, total PA, smoking habit, alcohol consumption and history of stroke, diabetes and heart disease.

## Discussion

The present study had the following main findings: (1) an inverse association between plant protein intake and sarcopenia was observed in males; (2) a U-shaped association was observed between animal-to-plant protein ratio and risk of sarcopenia; and (3) no association was observed between total and animal protein intakes and risk of sarcopenia.

In the present study, higher plant protein intake was associated with a lower risk of sarcopenia. To date, only one cohort study has examined the association between dietary plant protein intake and risk of sarcopenia. A cohort study by Li *et al.* ^(13)^, which followed 3380 Hong Kong residents (mean age, 72 years) for 4 years, found that the risk of sarcopenia was significantly lower in the highest tertile of energy-adjusted plant protein intake compared to the lowest tertile (Hazard ratio, 0·75), although total and animal protein intakes were not associated with sarcopenia. The present results are consistent with those of Li *et al.*’s study^(13)^.

Previous studies on longitudinal associations between plant protein intake and skeletal muscle mass or strength have yielded inconsistent findings. Li *et al.*’s cohort study mentioned above^(13)^ reported a smaller 4-year decline in appendicular skeletal muscle mass in individuals with higher plant protein intake. In a study of middle-aged and older Chinese adults (*n* 2709), Chen *et al.* ^(36)^ reported that a higher intake of energy-adjusted plant protein was associated with a smaller 3-year decline in ALM. In a study of older Americans (*n* 2066), Houston *et al.* ^(16)^ found no association between energy-adjusted plant protein intake and 3-year change in ALM. Moreover, in a study of older Americans (*n* 646), McLean *et al.* ^(37)^ found no association between energy-adjusted plant protein intake and 6-year change in grip strength. In another study of middle-aged and older Americans (*n* 1896), Yuan *et al.* ^(38)^ found no association between weight-adjusted plant protein intake and 14-year change in grip strength. Notably, the two studies^(13,36)^ conducted on East Asians, who consume relatively large amounts of plant protein from sources such as legumes, observed effects of plant protein intake on the prevention of muscle mass loss or sarcopenia.

Inflammation is a key factor in muscle protein degradation^(39)^, and it is thought that systemic low-grade chronic inflammation associated with ageing (inflammaging) contributes to the development of sarcopenia^(40)^. Inflammaging has been reported to upregulate proteasome activity through the induction of nuclear factor kappa-light-chain-enhancer of activated B cells signalling and protein oxidation, potentially damaging the insulin-dependent anabolic capacity of human skeletal muscle^(41)^. A recent meta-analysis of randomised controlled trials reported a reduction in serum TNF-*α* levels with soya protein supplementation^(42)^. In addition, mice fed isoflavone-free soya protein showed asuppressed inflammatory response to lipopolysaccharide, a component of the cell wall of gram-negative bacteria, compared to mice fed animal protein (casein)^(43)^. These results suggest that the intake of soya protein may be involved in the prevention of sarcopenia through its anti-inflammatory effects. In addition to inflammation, another proposed mechanism is that polyphenols found in plant protein-rich foods (such as grains, legumes, vegetables, fruits and nuts) may enhance antioxidant activity and beneficially modify the gut microbiota, potentially contributing to muscle mass maintenance^(44)^. Furthermore, according to one study, SCFA (butyrate) synthesis by gut microbiota has a beneficial effect on the host’s skeletal muscle mass^(45)^.

In general, legume proteins are rich in lysine but low in methionine and cysteine^(46)^, making them less efficient for protein anabolism compared to animal proteins. In East Asia, including Japan, rice is also a notable source of protein. A key characteristic of rice-derived protein is that it is low in lysine but high in methionine^(47)^. When rice and legumes are consumed in an appropriate ratio, the essential amino acid profile improves, enhancing protein anabolism efficiency. Differences in protein intake patterns, including dietary habits, may partly explain the discrepancies between studies conducted in Western populations and those in East Asia, including the present study. To better understand the effects of plant protein on sarcopenia, examining the specific composition of plant proteins may be necessary.

An inverse association between plant protein intake and sarcopenia was suggested in males. Although the reasons are not entirely clear, several plausible explanations can be considered. Circulating C-reactive protein levels^(48)^, which reflect systemic inflammation, and oxidative stress levels^(49)^ are known to be higher in Japanese males than in females. Hence, the intake of plant protein (i.e. soya protein and/or polyphenols) may exert greater benefits in males than in females. In addition, plant protein intake relative to energy intake among adults aged ≥ 40 years is lower in males according to the 2013 National Health and Nutrition Survey in Japan^(50)^. Furthermore, although males have greater muscle mass, the rate of muscle loss in middle-aged and older adult males is higher compared with their female counterparts^(51)^. Taken together, it is suggested that high plant protein intake may be more beneficial for males than females.

In the present study, a U-shaped association was found between animal-to-plant protein ratio (median: 0·82 for males and 0·90 for females) and risk of sarcopenia. *P*_for interaction_ for the U-shaped association by testing the interaction term involving the quadratic component was not significant (*P*_for interaction_ = 0·92, data not shown). Based on this result, we consider that the apparent difference in the U-shaped association between males and females is likely due to unstable estimates arising from the small number of sarcopenia cases; therefore, these findings should be interpreted with caution. The observed U-shaped association suggests that maintaining a balance between animal and plant protein intakes is crucial, and that protein intake skewed towards either is undesirable. Although the effectiveness of animal protein was not demonstrated in the present study, animal proteins are known to be of higher quality than plant proteins, as they are more easily digested and supply all essential amino acids necessary for stimulating muscle protein synthesis and preserving muscle integrity^(52)^. A U-shaped association between protein ratio and risk of sarcopenia has not been reported, especially in studies from Western countries. One reason for this could be that protein intake patterns differ markedly between Japanese people and Western populations. The mean animal-to-plant protein ratio was 0·95 for males and 1·06 for females in the present study, whereas the ratio was 1·2 for males and 1·1 for females among middle-aged and older adults participating in the 2013 National Health and Nutrition Survey^(50)^. In contrast, studies conducted in the USA reported much higher animal-to-plant protein ratios overall, with a ratio of 2·6 reported in one study^(37)^ and 2·8 in another^(53)^.

In the present study, total and animal protein intakes were not associated with risk of sarcopenia. Similar results were observed in a cohort study of older Hong Kong residents by Li *et al.* ^(13)^. The target population of the present study consisted of a physically active local population, and thus it is possible that the protein intake of most participants was generally adequate. The total protein intake per BW per day for the present study participants was 1·1 g/kg BW/d for males (interquartile range: 0·8, 1·4) and 1·3 g/kg BW/d for females (interquartile range: 1·0, 1·7). The protein maintenance requirements for middle-aged and older adults, based on the nitrogen balance method for Japanese people, are estimated to be 0·65–0·69 g/kg BW/d^(54)^. Therefore, it is possible that no association was observed between total protein intake and risk of sarcopenia due to the healthy study population.

The percentage of animal protein intake of the present study participants was clearly lower than that of Western populations. As mentioned above, studies in the USA reported animal-to-plant protein ratios of 2·6 to 2·8^(37,53)^, whereas the present study population had a ratio of approximately 1. The positive correlation observed between animal protein intake and skeletal muscle mass or muscle strength in the US study^(16,37,38)^ is likely due to the presence of a large number of individuals with high animal protein intake. If this is the case, increasing animal protein intake may reduce the risk of sarcopenia in Japanese people.

This study has some limitations. First, although the observation period was relatively long, averaging nine years, the presence or absence of sarcopenia at baseline was not assessed. This could have resulted in the inclusion of individuals who already had sarcopenia at study initiation. If so, the OR may have been affected by misclassification of cases. Second, although muscle strength was assessed, physical function was not, and thus not all sarcopenia cases may have been included. Since muscle mass and strength are major components of sarcopenia^(55)^, the specificity of this diagnostic method is considered high, but its sensitivity may have been slightly lower. Third, because protein intake was estimated based on a single baseline assessment using a self-administered questionnaire, it is possible that intake status changed during the follow-up period, leading to misclassification in the protein intake categories. Because stable dietary habits cannot be assumed over a 9-year period in real-world settings, longitudinal studies that incorporate long-term changes in dietary patterns are necessary to confirm our findings. Fourth, anthropometric characteristics and lifestyle factors were obtained through self-report. In such cases, differential misclassification bias may occur. For example, BW tends to be underreported, which may consequently lead to an underestimation of BMI. Fifth, protein intake assessed by the FFQ does not correlate well with intake measured by the dietary record method. Therefore, we may have failed to detect associations that might have been present. In particular, the validity of animal protein intake assessed by the FFQ (Spearman’s *r* = 0·34 in men and 0·23 in women) was lower than that of plant protein intake, especially in women. This may be attributable to the low validity of meat intake, which is the major contributor to animal protein intake^(20)^. Consequently, misclassification due to this low validity may have led to an underestimation of the association between animal protein intake and sarcopenia in this study. Sixth, younger individuals were less likely to participate in the follow-up examination. For this reason, the findings of this study are more likely to be applicable to the older subgroup within the middle-aged population. Seventh, residual confounding may remain. Important sources of such confounding include prolonged immobility due to hospitalisation and genetic factors. Finally, the findings of the present study should be interpreted with caution, given the relatively small number of sarcopenia cases. These limitations need to be addressed in future studies.

The present study has several public health implications. First, balanced protein intake is important. As a public health recommendation, an optimal range for the animal-to-plant protein ratio of 0·60–1·09 for males and 0·70–1·16 for females may be suggested based on the median values of the 2nd and 3rd quintiles (low-risk range). Second, balanced protein intake appears to differ between regions, that is, the USA and Japan (animal-to-plant protein ratio: approximately > 2 and 1, respectively). In this context, recommendations to ‘increase plant protein intake’ in the USA and to ‘maintain a balanced mix’ in Japan may be appropriate. Finally, plant protein intake may play an important role in preventing sarcopenia. However, since this association was observed only in males, further studies are warranted to confirm this relationship in females.

### Conclusions

The findings of this study highlight the importance of balancing animal and plant protein intakes, since an imbalance in the consumption of either may increase the risk of sarcopenia. Future studies to examine whether increasing animal protein intake can prevent sarcopenia in populations with low animal protein consumption are warranted.

## Supporting information

10.1017/S1368980026102973.sm001 Komata et al. supplementary material Komata et al. supplementary material

## Supplementary material

For supplementary material accompanying this paper visit https://doi.org/10.1017/S1368980026102973

## Table 1. Long description

The table presents participant characteristics at baseline according to quartiles of total, animal, and plant protein intakes for males. It includes 12 rows and 14 columns. The columns are labeled as follows: n, Age (years), BMI (kg/m^2), Total PA (MET-h/d), Energy (kcal/d), Total protein (g/d), Animal protein (g/d), Plant protein (g/d), Married (%), University graduates (%), Manual job (%), Current smoker (%), Current drinker (%), History of stroke (%), History of diabetes (%), and History of heart disease (%). The rows are labeled as Q1, Q2, Q3, and Q4, representing the quartiles of protein intake. Each row provides the median, interquartile range (IQR), and other relevant statistics for each characteristic. Notable trends include higher total protein intake associated with older age, lower physical activity levels, higher animal and plant protein intakes, and a higher likelihood of having a university degree. Additionally, higher animal protein intake is linked to lower physical activity levels, higher total protein intake, and a history of diabetes. Higher plant protein intake is associated with older age, higher total protein intake, and a lower likelihood of being smokers or drinkers.

Navigate back to Table 1..

## Table 2. Long description

The table presents participant characteristics at the 2011-2014 baseline survey according to quartiles of total, animal, and plant protein intakes in females. It includes data for 854 participants across four quartiles (Q1 to Q4). The table is divided into three main sections: Total protein, Animal protein, and Plant protein. Each section lists various characteristics such as age, BMI, total physical activity (PA), energy intake, protein intake, marital status, education level, job type, smoking status, drinking status, and medical history. For each characteristic, the median, interquartile range (IQR), and percentage are provided. Notable trends include increasing age, BMI, and protein intake across higher quartiles of total and animal protein, while plant protein intake shows a different distribution pattern. Education level and job type also vary significantly across quartiles. The table highlights how different levels of protein intake correlate with various participant characteristics.

Navigate back to Table 2..

## Table 3. Long description

The table presents data on the occurrence of sarcopenia across quartiles of energy-adjusted total protein intake for males, females, and combined. It includes unadjusted, age-adjusted, and multivariable-adjusted models for each group. The table has 12 rows and 11 columns. Column headers are Q1, Q2 OR, Q2 95 percent CI, Q3 OR, Q3 95 percent CI, Q4 OR, Q4 95 percent CI, and P for trend. Row labels are Males Occurrence of sarcopenia, Unadjusted, Age-adjusted, Multivariable-adjusted model 1, Multivariable-adjusted model 2, Females Occurrence of sarcopenia, Unadjusted, Age-adjusted, Multivariable-adjusted model 1, Multivariable-adjusted model 2, Males and females combined Occurrence of sarcopenia, Unadjusted, Age-adjusted, Multivariable-adjusted model 1, Multivariable-adjusted model 2. Each row provides specific data points for the occurrence of sarcopenia and odds ratios with confidence intervals. Notable trends include variations in the occurrence of sarcopenia and odds ratios across different quartiles and adjustment models.

Navigate back to Table 3..

## Table 4. Long description

The table presents data on the occurrence of sarcopenia across quartiles of energy-adjusted protein intake for males, females, and combined groups. It includes unadjusted, age-adjusted, and multivariable-adjusted models for both animal and plant protein intakes. The table has 20 rows and 10 columns. Column headers are Q1, Q2 OR, Q2 95% CI, Q3 OR, Q3 95% CI, Q4 OR, Q4 95% CI, and P for trend. Row labels include Animal protein males, Females, Males and females combined, and Plant protein males. Each row provides the occurrence of sarcopenia in percent and odds ratios (OR) with 95% confidence intervals (CI) for different models. Notable trends include higher plant protein intake being associated with lower odds of sarcopenia in males and the overall population, with significant P for trend values in multivariable-adjusted models.

Navigate back to Table 4..

## Table 5. Long description

The table presents data on the occurrence of sarcopenia across different quintiles of animal-to-plant protein ratio for males, females, and combined. It includes unadjusted, age-adjusted, and multivariable-adjusted models for each group. The table has 15 rows and 11 columns. Column headers are Q1, Q2 OR, Q2 95% CI, Q3 OR, Q3 95% CI, Q4 OR, Q4 95% CI, Q5 OR, Q5 95% CI, and P for quadratic term. Row labels include Males, Females, and Males and females combined, with sub-rows for Occurrence of sarcopenia, Unadjusted, Age-adjusted, Multivariable-adjusted model 1, and Multivariable-adjusted model 2. Each row provides specific values for the occurrence of sarcopenia and odds ratios (OR) with 95% confidence intervals (CI) for each quintile. Notable trends include higher odds ratios for sarcopenia in the 5th quintile for males and the 1st quintile for females compared to the middle quintile (reference). The overall population shows similar patterns with higher odds ratios in the 1st and 5th quintiles.

Navigate back to Table 5..
