# Supplementary material for: Dietary protein intake, animal-to-plant protein ratio and risk of sarcopenia in community-dwelling adults: a 9-year follow-up study
Source: Public Health Nutr. 2026 Jul 2;29(1):e153. doi: 10.1017/S1368980026102973 (PMC13434553; doi:10.1017/S1368980026102973)
Supplement: Supplementary file 1 [file S1368980026102973sup001.docx]

Supplementary Table 1. Codes for demographic variables, smoking, and alcohol consumption

Variables

Marital status	(a) married, (b) never married, and (c) divorced, separated, or bereaved

Education level	(1) junior high school, (2) high school, (3) junior or vocational college, and (4) university graduates

Occupation	(a) office work and sales/service work, (b) professional/management, (c) manual (security, farming/forestry/fishery, transportation, and labor services), and (d) no job/others

Smoking	(1) non-smoker, (2) past smoker, (3) 1–19 cigarettes/day, and (4) ≥20 cigarettes/day

Alcohol consumption	(1) non- or rare drinker, (2) 1–149 g ethanol/week, (3) 150–299 g ethanol/week,

(4) 300–449 g ethanol/week, and (5) ≥450 g ethanol/week

Supplementary Table 2. Differences in percent frequencies of age and sex at baseline between the subsample and total sample of the two cohorts.

Subsample	Total sample	Difference in percent frequency^*^

(N = 6,971)	(N = 45,071)

Age (years)

40–49	444 (6.4%)	9,064 (20.1%)	−13.7%

50–59	1,538 (22.1%)	12,972 (28.8%)	−6.7%

60–69	3,822 (54.8%)	16,632 (36.9%)	17.9%

70–74	1,167 (16.7%)	6,403 (14.2%)	2.5%

Sex

Males	3,177 (45.6%)	21,880 (48.5%)	−3.0%

Females	3,794 (54.4%)	23,191 (51.5%)	3.0%

Education level

Junior high school	2,418 (35.4%)	13,472 (30.6%)	4.7%

High school	3,100 (45.3%)	20,500 (46.6%)	−1.3%

Junior or vocational college	945 (13.8%)	7,027 (16.0%)	−2.2%

University	376 (5.5%)	2,974 (6.8%)	−1.3%

Occupation

Office work and sales/service work	1,380 (20.1%)	10,307 (23.2%)	−3.2%

Professional/management	968 (14.1%)	9,151 (20.6%)	−6.5%

Manual	1,555 (22.6%)	9,879 (22.2%)	0.4%

No job/others	2,979 (43.3%)	15,078 (33.9%)	9.3%

^*^Percent frequency of the total sample subtracted from percent frequency of the subsample.

All differences were significant at P < 0.05 based on the chi-square test, except for manual occupation.

Supplementary Table 3. Participant characteristics at the 2011–2014 baseline survey according to quartiles of total, animal, and plant protein intakes in males and females combined.

Quartiles of energy-adjusted intake

Q1	Q2	Q3	Q4	P for trend

Total protein

N	1558	1559	1558	1557

Age (years)	62.0 (57.0, 66.0)	63.0 (59.0, 67.0)	64.0 (59.0, 68.0)	64.0 (60.0, 68.0)	<0.001

BMI (kg/m^2^)	22.4 (20.5, 24.2)	22.4 (20.8, 24.3)	22.5 (20.7, 24.1)	22.5 (20.6, 24.4)	0.11

Total PA (MET-h/d)	40.8 (35.3, 51.0)	40.2 (35.0, 48.4)	40.2 (35.2, 48.6)	39.4 (35.1, 47.4)	0.004

Energy (kcal/d)	1992 (1607, 2432)	2051 (1645, 2465)	1972 (1629, 2405)	1968 (1564, 2423)	0.31

Total protein (g/d)	53.4 (41.2, 67.7)	65.3 (51.3, 82.4)	69.5 (56.1, 88.2)	79.6 (62.6, 105.5)	<0.001

Animal protein (g/d)	19.3 (12.5, 27.5)	28.1 (19.9, 38.8)	33.5 (24.3, 46.5)	43.6 (30.7, 63.6)	<0.001

Plant protein (g/d)	32.9 (26.3, 41.9)	35.9 (29.2, 44.6)	35.4 (28.9, 43.1)	33.4 (27.4, 42.1)	0.048

Married (%)	1273 (81.7 %)	1312 (84.2 %)	1318 (84.6 %)	1278 (82.1 %)	0.67

University graduates (%)	58 (3.7 %)	81 (5.2 %)	105 (6.7 %)	109 (7.0 %)	<0.001

Manual job (%)	398 (25.5 %)	350 (22.5 %)	361 (23.2 %)	274 (17.6 %)	<0.001

Current smoker (%)	279 (17.9 %)	206 (13.2 %)	184 (11.8 %)	182 (11.7 %)	<0.001

Current drinker (%)	1062 (68.2 %)	955 (61.3 %)	901 (57.8 %)	790 (50.7 %)	<0.001

History of stroke (%)	20 (1.3 %)	29 (1.9 %)	35 (2.3 %)	35 (2.3 %)	0.042

History of diabetes (%)	76 (4.9 %)	92 (5.9 %)	83 (5.3 %)	119 (7.6 %)	0.003

History of heart disease (%)	6 (0.4 %)	11 (0.7 %)	13 (0.8 %)	7 (0.5 %)	0.74

Animal protein

N	1557	1560	1558	1557

Age (years)	63.0 (58.0, 67.0)	63.0 (58.0, 67.0)	64.0 (59.0, 68.0)	64.0 (59.0, 68.0)	<0.001

BMI (kg/m^2^)	22.5 (20.6, 24.4)	22.4 (20.8, 24.2)	22.4 (20.7, 24.2)	22.5 (20.6, 24.4)	0.38

Total PA (MET-h/d)	40.9 (35.4, 50.1)	40.6 (35.4, 49.1)	39.9 (35.2, 48.4)	39.3 (34.8, 47.7)	0.001

Energy (kcal/d)	2005 (1583, 2443)	2038 (1679, 2485)	1999 (1630, 2413)	1920 (1536, 2388)	0.012

Total protein (g/d)	56.0 (43.0, 72.6)	65.0 (51.5, 83.2)	68.9 (56.0, 87.7)	75.5 (58.9, 102.7)	<0.001

Animal protein (g/d)	16.8 (11.4, 23.9)	27.8 (20.7, 37.5)	34.9 (25.9, 47.5)	45.0 (32.5, 65.7)	<0.001

Plant protein (g/d)	38.4 (30.3, 48.4)	37.0 (30.1, 45.0)	34.2 (28.6, 40.8)	30.0 (24.4, 35.6)	<0.001

Married (%)	1280 (82.2 %)	1322 (84.7 %)	1292 (82.9 %)	1287 (82.7 %)	0.80

University graduates (%)	56 (3.6 %)	77 (4.9 %)	119 (7.6 %)	101 (6.5 %)	<0.001

Manual job (%)	369 (23.7 %)	383 (24.6 %)	351 (22.5 %)	280 (18.0 %)	<0.001

Current smoker (%)	260 (16.7 %)	215 (13.8 %)	178 (11.4 %)	198 (12.7 %)	<0.001

Current drinker (%)	974 (62.6 %)	953 (61.1 %)	926 (59.4 %)	855 (54.9 %)	<0.001

History of stroke (%)	31 (2.0 %)	26 (1.7 %)	26 (1.7 %)	36 (2.3 %)	0.51

History of diabetes (%)	85 (5.5 %)	80 (5.1 %)	83 (5.3 %)	122 (7.8 %)	0.004

History of heart disease (%)	8 (0.5 %)	7 (0.5 %)	11 (0.7 %)	11 (0.7 %)	0.34

Plant protein

N	1557	1560	1557	1558

Age (years)	63.0 (57.0, 66.0)	63.0 (58.0, 67.0)	64.0 (59.0, 68.0)	64.0 (60.0, 68.0)	<0.001

BMI (kg/m^2^)	22.4 (20.6, 24.2)	22.6 (20.7, 24.3)	22.5 (20.7, 24.2)	22.4 (20.6, 24.3)	0.40

Total PA (MET-h/d)	39.9 (34.9, 49.2)	39.2 (34.8, 48.5)	40.3 (35.4, 48.5)	41.0 (35.6, 49.0)	0.41

Energy (kcal/d)	2001 (1580, 2541)	1934 (1587, 2335)	1972 (1604, 2415)	2067 (1656, 2481)	0.011

Total protein (g/d)	65.4 (47.8, 92.6)	63.9 (49.5, 80.3)	65.0 (51.6, 84.1)	70.8 (56.4, 89.1)	<0.001

Animal protein (g/d)	38.5 (24.2, 60.4)	31.1 (21.5, 44.1)	28.5 (19.2, 41.1)	24.9 (17.0, 35.1)	<0.001

Plant protein (g/d)	27.0 (21.9, 32.7)	32.0 (27.5, 37.7)	36.8 (31.0, 43.8)	44.9 (37.2, 53.9)	<0.001

Married (%)	1261 (81.0 %)	1319 (84.6 %)	1296 (83.2 %)	1305 (83.8 %)	0.045

University graduates (%)	93 (6.0 %)	81 (5.2 %)	95 (6.1 %)	84 (5.4 %)	0.75

Manual job (%)	337 (21.6 %)	354 (22.7 %)	354 (22.7 %)	338 (21.7 %)	0.98

Current smoker (%)	263 (16.9 %)	214 (13.7 %)	186 (12.0 %)	188 (12.1 %)	<0.001

Current drinker (%)	1034 (66.4 %)	963 (61.7 %)	912 (58.6 %)	799 (51.3 %)	<0.001

History of stroke (%)	23 (1.5 %)	25 (1.6 %)	32 (2.1 %)	39 (2.5 %)	0.022

History of diabetes (%)	98 (6.3 %)	85 (5.5 %)	86 (5.5 %)	101 (6.5 %)	0.81

History of heart disease (%)	11 (0.7 %)	7 (0.5 %)	11 (0.7 %)	8 (0.5 %)	0.71

PA, physical activity; MET, metabolic equivalent.

Median with interquartile range in parentheses or number. BMI, energy intake, total protein intake, animal protein intake, and plant protein intake were log-transformed when calculating P for trend.

Supplementary Table 4. Participant characteristics at the 2011–2014 baseline survey according to quintiles of animal-to-plant protein ratio

Quintiles of animal-to-plant protein ratio

Q1	Q2	Q3	Q4	Q5	P for trend

Males

N	563	563	563	563	563

Age (years)	64.0 (60.0, 68.0)	63.0 (60.0, 67.0)	64.0 (60.0, 69.0)	64.0 (59.0, 68.0)	64.0 (59.0, 69.0)	0.90

BMI (kg/m^2^)	22.9 (21.1, 24.6)	22.9 (21.5, 24.6)	22.8 (21.0, 24.4)	23.0 (21.3, 24.6)	23.1 (21.3, 24.8)	0.45

Total PA (MET-h/d)	42.2 (35.1, 52.2)	42.4 (35.2, 51.9)	41.8 (35.9, 52.1)	41.7 (35.4, 52.3)	41.9 (34.9, 53.8)	0.32

Energy (kcal/d)	1899 (1543, 2337)	2060 (1729, 2441)	2101 (1789, 2499)	2233 (1872, 2675)	2453 (2016, 3012)	<0.001

Total protein (g/d)	49.1 (36.9, 62.6)	59.8 (47.4, 71.1)	66.0 (52.6, 79.7)	73.6 (60.7, 91.2)	90.2 (72.0, 116)	<0.001

Animal protein (g/d)	12.5 (7.6, 16.7)	21.9 (17.8, 26.8)	29.5 (23.4, 36.0)	38.2 (31.3, 48.1)	57.4 (43.9, 74.8)	<0.001

Plant protein (g/d)	36.4 (28.8, 46.6)	37.4 (29.8, 44.7)	36.3 (29.3, 44.4)	34.8 (28.7, 43.3)	32.2 (26.1, 39.1)	<0.001

Animal to plant protein ratio	0.3 (0.2, 0.4)	0.6 (0.5, 0.7)	0.8 (0.8, 0.9)	1.1 (1.0, 1.2)	1.7 (1.4, 2.1)	<0.001

Married (%)	461 (81.9 %)	471 (83.7 %)	462 (82.1 %)	490 (87.0 %)	477 (84.7 %)	0.08

University graduates (%)	31 (5.5 %)	49 (8.7 %)	50 (8.9 %)	71 (12.6 %)	61 (10.8 %)	<0.001

Manual job (%)	191 (33.9 %)	199 (35.3 %)	203 (36.1 %)	182 (32.3 %)	168 (29.8 %)	0.08

Current smoker (%)	163 (29.0 %)	136 (24.2 %)	136 (24.2 %)	133 (23.6 %)	133 (23.6 %)	0.0495

Current drinker (%)	460 (81.7 %)	444 (78.9 %)	465 (82.6 %)	490 (87.0 %)	466 (82.8 %)	0.041

History of stroke (%)	19 (3.4 %)	14 (2.5 %)	16 (2.8 %)	16 (2.8 %)	11 (2.0 %)	0.25

History of diabetes (%)	43 (7.6 %)	45 (8.0 %)	35 (6.2 %)	59 (10.5 %)	59 (10.5 %)	0.025

History of heart disease (%)	8 (1.4 %)	8 (1.4 %)	5 (0.9 %)	8 (1.4 %)	6 (1.1 %)	0.63

Females

N	684	683	683	683	684

Age (years)	62.0 (58.0, 66.0)	62.0 (57.0, 67.0)	63.0 (58.0, 67.0)	63.0 (58.0, 67.0)	63.0 (59.0, 67.0)	0.030

BMI (kg/m^2^)	21.8 (20.0, 24.0)	22.0 (20.2, 24.0)	21.9 (20.4, 23.7)	22.2 (20.4, 23.9)	22.2 (20.3, 24.1)	0.025

Total PA (MET-h/d)	39.0 (34.8, 47.1)	39.0 (35.2, 45.7)	39.0 (35.3, 45.8)	39.2 (35.2, 46.4)	39.2 (34.8, 46.6)	0.83

Energy (kcal/d)	1645 (1336, 2064)	1728 (1454, 2139)	1829 (1532, 2180)	1934 (1613, 2317)	2190 (1773, 2759)	<0.001

Total protein (g/d)	51.1 (41.1, 65.4)	58.7 (48.5, 74.4)	64.6 (53.3, 80.1)	72.1 (59.5, 89.6)	92.8 (71.9, 118.9)	<0.001

Animal protein (g/d)	15.1 (11.3, 20.4)	24.1 (19.6, 30.3)	30.6 (25.0, 38.0)	39.1 (31.9, 48.6)	59.2 (45.4, 79.1)	<0.001

Plant protein (g/d)	36.1 (28.8, 45.8)	34.8 (28.7, 44.2)	34.1 (28.0, 42.4)	33.5 (27.5, 41.1)	31.9 (25.7, 38.2)	<0.001

Animal to plant protein ratio	0.4 (0.3, 0.5)	0.7 (0.6, 0.7)	0.9 (0.9, 1.0)	1.2 (1.1, 1.2)	1.8 (1.5, 2.3)	<0.001

Married (%)	567 (82.9 %)	570 (83.5 %)	546 (79.9 %)	563 (82.4 %)	574 (83.9 %)	0.91

University graduates (%)	6 (0.9 %)	19 (2.8 %)	21 (3.1 %)	21 (3.1 %)	24 (3.5 %)	0.008

Manual job (%)	102 (14.9 %)	89 (13.0 %)	87 (12.7 %)	70 (10.2 %)	92 (13.5 %)	0.15

Current smoker (%)	43 (6.3 %)	27 (4.0 %)	34 (5.0 %)	21 (3.1 %)	25 (3.7 %)	0.011

Current drinker (%)	248 (36.3 %)	279 (40.9 %)	287 (42.0 %)	280 (41.0 %)	289 (42.3 %)	0.042

History of stroke (%)	10 (1.5 %)	7 (1.0 %)	6 (0.9 %)	12 (1.8 %)	8 (1.2 %)	0.91

History of diabetes (%)	29 (4.2 %)	22 (3.2 %)	24 (3.5 %)	25 (3.7 %)	29 (4.2 %)	0.84

History of heart disease (%)	0 (0.0 %)	0 (0.0 %)	1 (0.2 %)	0 (0.0 %)	1 (0.2 %)	0.34

Males and females combined

N	1247	1246	1246	1246	1247

Age (years)	63.0 (59.0, 67.0)	63.0 (58.0, 67.0)	63.0 (59.0, 67.0)	63.0 (58.0, 67.0)	64.0 (59.0, 68.0)	0.09

BMI (kg/m^2^)	22.4 (20.5, 24.3)	22.5 (20.7, 24.3)	22.2 (20.7, 24.1)	22.6 (20.8, 24.2)	22.6 (20.7, 24.5)	0.029

Total PA (MET-h/d)	40.4 (34.9, 49.2)	40.2 (35.2, 48.3)	39.9 (35.6, 48.4)	40.0 (35.4, 48.6)	40.2 (34.8, 49.5)	0.38

Energy (kcal/d)	1728 (1415, 2178)	1876 (1554, 2299)	1952 (1606, 2333)	2091 (1732, 2493)	2315 (1877, 2903)	<0.001

Total protein (g/d)	50.2 (39.2, 64.4)	59.1 (48.2, 72.7)	65.2 (52.7, 79.8)	72.8 (60.0, 90.5)	92.1 (72.0, 117.4)	<0.001

Animal protein (g/d)	14.0 (9.5, 18.7)	23.0 (18.8, 28.7)	30.1 (24.1, 37.1)	38.7 (31.7, 48.3)	58.5 (44.6, 76.5)	<0.001

Plant protein (g/d)	36.2 (28.8, 46.4)	36.0 (29.2, 44.3)	35.0 (28.3, 43.4)	34.2 (28.1, 42.1)	32.0 (26.0, 38.6)	<0.001

Animal to plant protein ratio	0.4 (0.3, 0.5)	0.7 (0.6, 0.7)	0.9 (0.8, 0.9)	1.1 (1.1, 1.2)	1.7 (1.5, 2.2)	<0.001

Married (%)	1028 (82.4 %)	1041 (83.5 %)	1008 (80.9 %)	1053 (84.5 %)	1051 (84.3 %)	0.22

University graduates (%)	37 (3.0 %)	68 (5.5 %)	71 (5.7 %)	92 (7.4 %)	85 (6.8 %)	<0.001

Manual job (%)	293 (23.5 %)	288 (23.1 %)	290 (23.3 %)	252 (20.2 %)	260 (20.9 %)	0.029

Current smoker (%)	206 (16.5 %)	163 (13.1 %)	170 (13.6 %)	154 (12.4 %)	158 (12.7 %)	0.005

Current drinker (%)	708 (56.8 %)	723 (58.0 %)	752 (60.4 %)	770 (61.8 %)	755 (60.6 %)	0.010

History of stroke (%)	29 (2.3 %)	21 (1.7 %)	22 (1.8 %)	28 (2.3 %)	19 (1.5 %)	0.40

History of diabetes (%)	72 (5.8 %)	67 (5.4 %)	59 (4.7 %)	84 (6.7 %)	88 (7.1 %)	0.06

History of heart disease (%)	8 (0.6 %)	8 (0.6 %)	6 (0.5 %)	8 (0.6 %)	7 (0.6 %)	0.82

PA, physical activity; MET, metabolic equivalent.

Median with interquartile range in parentheses or number. BMI, energy intake, total protein intake, animal protein intake, plant protein intake, and animal-to-plant protein ratio were log-transformed when calculating P for trend.

Cutoff values for quintiles of animal-to-plant protein ratio were 0.49, 0.70, 0.94, and 1.30 for males, and 0.58, 0.80, 1.02, and 1.38 for females.

Supplementary Table 5. Results of the 2021–2022 physical examinations by sex

Males (N= 2815)	Females (N= 3417)

ALM (kg)	20.5 (18.3, 22.8)	14.9 (13.6, 16.2)

Adjusted ALM by height squared (kg/m^2^)	7.7 (7.1, 8.2)	6.5 (6.2, 6.8)

Grip strength (kg)	37.3 (33.0, 41.6)	24.3 (21.7, 26.9)

Low adjusted ALM^*^	554 (19.7%)	673 (19.7%)

Low grip strength^†^	203 (7.2%)	195 (5.7%)

Sarcopenia^‡^	135 (4.8%)	122 (3.6%)

ALM, appendicular lean mass.

^*^Cutoff values of adjusted ALM are <7.0 kg/m^2^ for males and <6.1 kg/m^2^ for females.

^†^Cutoff values of grip strength are <28 kg for males and <18 kg for females.

Median with interquartile range in parentheses or number.

^‡^Sarcopenia was defined as having both low adjusted ALM^*^ and low grip strength^†^.

Supplementary Table 6. Odds ratios (ORs) for sarcopenia according to quartiles of plant protein intake in participants with no disease history (sensitivity analysis, N=5731)

Quartiles of energy-adjusted plant protein intake	P for trend

Q1	Q2	Q3	Q4

Males

Occurrence of sarcopenia	30/620 (4.8%)	26/620 (4.2%)	21/624 (3.4%)	28/619 (4.5%)

Unadjusted OR (95% CI)	1 (ref)	0.86 (0.50-1.47)	0.69 (0.39-1.21)	0.93 (0.55-1.58)	0.62

Age-adjusted OR (95% CI)	1 (ref)	0.74 (0.42-1.29)	0.48 (0.27-0.86)	0.67 (0.39-1.16)	0.08

Multivariable-adjusted model 1 OR^*^ (95% CI)	1 (ref)	0.70 (0.39-1.25)	0.44 (0.23-0.84)	0.60 (0.31-1.14)	0.07

Multivariable-adjusted model 2 OR^†^ (95% CI)	1 (ref)	0.76 (0.42-1.37)	0.47 (0.24-0.91)	0.60 (0.31-1.16)	0.08

Females

Occurrence of sarcopenia	26/810 (3.2%)	30/826 (3.6%)	21/809 (2.6%)	35/803 (4.4%)

Unadjusted OR (95% CI)	1 (ref)	1.14 (0.67-1.94)	0.80 (0.45-1.44)	1.37 (0.82-2.31)	0.41

Age-adjusted OR (95% CI)	1 (ref)	1.08 (0.63-1.85)	0.71 (0.39-1.27)	1.17 (0.69-1.98)	0.85

Multivariable-adjusted model 1 OR^*^ (95% CI)	1 (ref)	1.04 (0.59-1.83)	0.65 (0.35-1.22)	1.09 (0.58-2.04)	0.89

Multivariable-adjusted model 2 OR^†^ (95% CI)	1 (ref)	0.98 (0.55-1.74)	0.65 (0.35-1.23)	1.03 (0.55-1.93)	0.80

Males and females combined

Occurrence of sarcopenia	56/1430 (3.9%)	56/1446 (3.9%)	42/1433 (2.9%)	63/1422 (4.4%)

Unadjusted OR (95% CI)	1 (ref)	0.99 (0.68-1.44)	0.74 (0.49-1.11)	1.14 (0.79-1.64)	0.79

Age-adjusted OR (95% CI)	1 (ref)	0.90 (0.61-1.33)	0.60 (0.39-0.90)	0.91 (0.62-1.32)	0.32

Multivariable-adjusted model 1 OR^‡^ (95% CI)	1 (ref)	0.87 (0.58-1.29)	0.55 (0.36-0.86)	0.80 (0.52-1.24)	0.15

Multivariable-adjusted model 2 OR^†^ (95% CI)	1 (ref)	0.87 (0.58-1.30)	0.56 (0.36-0.88)	0.76 (0.49-1.19)	0.11

^*^Adjusted for age, marital status, education, occupation, energy-adjusted animal protein intake, total physical activity (PA), smoking habit, and alcohol consumption.

^†^Further adjusted for log-transformed BMI in addition to the variables included in multivariable-adjusted model 1.

^‡^Adjusted for sex, age, marital status, education, occupation, energy-adjusted animal protein intake, total PA, smoking habit, and alcohol consumption.

Supplementary Table 7. Odds ratios (ORs) for sarcopenia according to quintiles of animal-to-plant protein ratio in participants with no disease history (sensitivity analysis, N=5731)

Quintiles of animal-to-plant protein ratio	P for

Q1	Q2	Q3	Q4	Q5	quadratic term

Males

Occurrence of sarcopenia	25/496 (5.0%)	15/502 (3.0%)	18/512 (3.5%)	20/482 (4.1%)	27/491 (5.5%)

Unadjusted OR (95% CI)	1.46 (0.79-2.71)	0.85 (0.42-1.70)	1 (ref)	1.19 (0.62-2.27)	1.60 (0.87-2.94)	0.043

Age-adjusted OR (95% CI)	1.72 (0.91-3.25)	1.10 (0.54-2.23)	1 (ref)	1.37 (0.70-2.66)	1.81 (0.97-3.38)	0.028

Multivariable-adjusted model 1 OR^*^ (95% CI)	1.61 (0.85-3.07)	1.16 (0.57-2.38)	1 (ref)	1.47 (0.75-2.88)	1.89 (1.00-3.57)	0.047

Multivariable-adjusted model 2 OR^†^ (95% CI)	1.80 (0.93-3.46)	1.30 (0.63-2.70)	1 (ref)	1.62 (0.81-3.23)	2.17 (1.13-4.17)	0.023

Females

Occurrence of sarcopenia	28/648 (4.3%)	17/655 (2.6%)	18/652 (2.8%)	22/647 (3.4%)	27/646 (4.2%)

Unadjusted OR (95% CI)	1.59 (0.87-2.91)	0.94 (0.48-1.84)	1 (ref)	1.24 (0.66-2.33)	1.54 (0.84-2.82)	0.042

Age-adjusted OR (95% CI)	1.80 (0.98-3.32)	0.97 (0.49-1.92)	1 (ref)	1.28 (0.67-2.42)	1.51 (0.82-2.79)	0.029

Multivariable-adjusted model 1 OR^*^ (95% CI)	1.78 (0.96-3.30)	0.97 (0.49-1.93)	1 (ref)	1.28 (0.67-2.44)	1.58 (0.85-2.94)	0.025

Multivariable-adjusted model 2 OR^†^ (95% CI)	1.77 (0.94-3.31)	0.95 (0.48-1.90)	1 (ref)	1.36 (0.71-2.62)	1.71 (0.92-3.20)	0.020

Males and females combined

Occurrence of sarcopenia	53/1144 (4.6%)	32/1157 (2.8%)	36/1164 (3.1%)	42/1129 (3.7%)	54/1137 (4.7%)

Unadjusted OR (95% CI)	1.52 (0.99-2.34)	0.89 (0.55-1.45)	1 (ref)	1.21 (0.77-1.90)	1.56 (1.02-2.40)	0.004

Age-adjusted OR (95% CI)	1.76 (1.13-2.74)	1.01 (0.62-1.65)	1 (ref)	1.31 (0.83-2.08)	1.63 (1.05-2.53)	0.002

Multivariable-adjusted model 1 OR^‡^ (95% CI)	1.67 (1.08-2.61)	1.03 (0.63-1.68)	1 (ref)	1.37 (0.86-2.18)	1.69 (1.09-2.62)	0.003

Multivariable-adjusted model 2 OR^†^ (95% CI)	1.75 (1.12-2.75)	1.07 (0.65-1.77)	1 (ref)	1.49 (0.93-2.39)	1.89 (1.21-2.97)	0.001

^*^Adjusted for age, marital status, education, occupation, total physical activity (PA), smoking habit, and alcohol consumption.

^†^Further adjusted for log-transformed BMI in addition to the variables included in multivariable-adjusted model 1.

^‡^Adjusted for sex, age, marital status, education, occupation, total PA, smoking habit, and alcohol consumption.

Cutoff values for quintiles are 0.49, 070, 0.94, and 1.30 for males, and 0.58, 0.80, 1.02, and 1.38 for females.

Median values for quintiles are 0.34, 0.60, 0.82, 1.09, and 1.67 for males, and 0.44, 0.70, 0.90, 1.16, and 1.77 for females.
